# From Cerebrovascular Injury to Vascular Cognitive Impairment and Dementia: Therapeutic Potential of Stem Cell-Derived Extracellular Vesicles

**DOI:** 10.3390/biomedicines14010163

**Published:** 2026-01-13

**Authors:** Smara Sigdel, Harshal Sawant, Brandon Xiang Yu, Annie Chen, Rakan Albalawy, Jinju Wang

**Affiliations:** 1 Department of Biomedical Sciences, Joan C Edwards School of Medicine, Marshall University, Huntington, WV 25755, USA; 2 Department of Internal Medicine, Joan C Edwards School of Medicine, Marshall University, Huntington, WV 25755, USA

**Keywords:** extracellular vesicles, stem cells, vascular cognitive impairment and dementia, vascular dysfunction, cerebrovascular integrity impairment, BBB dysfunction

## Abstract

Vascular cognitive impairment and dementia (VCID) encompass a spectrum of cognitive syndromes ranging from mild cognitive impairment to vascular dementia, accounting for approximately 15–20% of all dementia cases and representing the second most common form of dementia. Despite its high prevalence and clinical burden, effective therapeutic strategies remain lacking. Increasing evidence indicates that vascular dysfunction plays a central role in the pathogenesis of VCID by compromising cerebrovascular integrity, impairing endothelial function, and disrupting neurovascular coupling, which collectively contribute to cognitive decline. Stem cells have emerged as promising candidates for promoting vascular repair and neurovascular coupling. Notably, extracellular vesicles (EVs) derived from stem cells exert reparative and protective effects by transferring bioactive molecules that enhance endothelial function and preserve the blood–brain barrier (BBB) function to affected regions. This review summarizes the current knowledge of VCID from a vascular perspective, highlights recent advances in understanding stem cells and their derived EVs in promoting vascular repair and alleviating cognitive decline, and discusses future directions for translating these insights into innovative therapeutic strategies for VCID.

## 1. Introduction

Vascular cognitive impairment and dementia (VCID) is composed of a continuum of cognitive disorders ranging from mild cognitive impairment to vascular dementia. It represents the second most common form of dementia after Alzheimer’s disease, accounting for approximately 15–20% of all cases worldwide [1]. The global prevalence of VCID is projected to reach 152.8 million by 2050 [2]. In addition to chronological aging, several vascular risk factors, including hypertension, diabetes, and hyperlipidemia, are strongly implicated in the pathogenesis of VCID. Among VCID patients, 79.4% had two vascular comorbidities, 62.3% had three vascular comorbidities, and 45.5% had four vascular comorbidities [3]. Furthermore, among these comorbidities, 65.9% of patients with a vascular dementia diagnosis had hypertension, 20.3% had diabetes, and 25.3% had suffered from a stroke [3]. In the United States, data from the Atherosclerosis Risk in Communities (ARIC) study, conducted by Markus and Joutel, demonstrated that individuals with cerebral small vessel disease, including white matter hyperintensity burden, lacune lesions, and microvessel rarefaction, had a 2–3-fold increased risk of developing post-stroke cognitive impairment and dementia [4]. Longitudinal studies show that midlife hypertension significantly increases the risk of developing dementia later in life [5,6]. Despite progress in managing vascular risk factors, effective treatments for VCID remain unavailable. Therefore, a deeper understanding of the vascular mechanisms underlying VCID pathophysiology is critical for developing novel therapeutic strategies.

In recent years, stem and progenitor cells have garnered increasing attention in regenerative medicine [7]. Stem cells, including endothelial progenitor cells (EPCs) [8,9], mesenchymal stem cells (MSCs) [10], and pericytes which have stem cell potential [11,12], are of particular interest due to their ability to maintain vascular homeostasis and promote vascular repair. Growing evidence indicates that many of the regenerative and protective functions of these stem cells are mediated not only by direct cellular activity, but also through their secreted extracellular vesicles (EVs) [13,14].

EVs are nanosized, membrane-bound vesicles that facilitate intercellular communication by transferring bioactive molecules, including microRNAs (miRs), proteins, lipids, and mitochondrial components, to recipient cells, and thereby modulating recipients’ functions [15]. Because EVs can freely cross the blood–brain barrier (BBB) and circulate systemically, they have emerged as promising therapeutic vehicles for neurological and vascular disorders. Numerous preclinical studies have shown that stem and progenitor cell-derived EVs enhance endothelial survival, promote angiogenesis, and preserve vascular integrity in models of stroke [13] and myocardial infarction [14]. These effects are largely attributed to the delivery of pro-regenerative miRs such as miR-21, miR-124, miR-132, miR-210, miR-150 [16], miR-26a [17], and proteins, including VEGF, IGF-1, PDGF that activate the angiogenic and anti-apoptotic signaling pathways. Our previous work demonstrated that EPC-derived EVs protect the brain against ischemic injury by promoting collateral circulation in the acute phase and stimulating angiogenesis during the chronic phase of diabetic ischemic stroke, primarily through miR-126-mediated mechanisms [13]. Other studies have reported that EVs modulate endothelial and pericyte functions by enhancing angiogenesis, stabilizing tight junctions, and reducing BBB leakage [18]. In addition, EVs carry anti-inflammatory cargo, such as miR-146a, and cytokines including TGFβ, HSP70, IL-10, which reprogram microglia and endothelial cells toward reparative phenotypes, thereby attenuating neurovascular inflammation [19]. Excitingly, recent preclinical studies revealed that EVs derived from young donors possess remarkable rejuvenating properties in aging models [20,21,22]. Treatment with EVs from young cardiosphere-derived cells improved the structural and functional integrity of multiple organs, including the heart, lungs, skeletal muscle, and kidneys, while enhancing glucose metabolism and activating anti-senescence pathways [20]. Similarly, intravenous administration of small EVs from young mice extended lifespan, reduced cellular senescence, and ameliorated age-associated functional decline across multiple tissues [22]. Collectively, these findings provide strong mechanistic support for the use of stem and progenitor cell-derived EVs to counteract premature vascular aging, preserve BBB function, and ultimately prevent or delay the progression of cognitive decline in VCID.

This review summarizes the pathogenic mechanisms of VCID from a vascular perspective, with emphasis on vascular dysfunction arising from premature vascular aging, BBB disruption, and mitochondrial injury. We further discuss the therapeutic potential of stem cell-derived EVs, explore their mechanisms of action in restoring vascular homeostasis, and highlight future directions and translational challenges for their application in VCID management.

## 2. Probable Pathogenic Mechanisms and Current Available Treatments for VCID

The precise pathogenic mechanisms underlying VCID remain incompletely understood. Nevertheless, growing clinical and experimental evidence indicates that vascular pathologies represent a central driver of disease progression [23,24]. These alterations are often initiated or accelerated by vascular aging and are further aggravated by vascular risk factors, including hypertension, diabetes, and hyperlipidemia [23,25]. Vascular aging [26], characterized by endothelial senescence and reduced nitric oxide (NO) availability, plays a pivotal mechanistic role in promoting arterial stiffening and compromising BBB integrity. In addition, mitochondrial injury in cerebrovascular endothelial cells induces excessive production of reactive oxygen species (ROS), which contribute to neurovascular uncoupling. Microvascular rarefaction further results in chronic cerebral hypoperfusion, impairing the neurovascular unit function and exacerbating white matter damage and progressive cognitive decline [27]. Collectively, these vascular and metabolic disturbances converge to promote astrocyte and microglial activation (reactive gliosis), neuroinflammation, and disruption of the neuro-glia communication. Overtime, these interlinked vascular and neuroinflammatory processes cause neuronal death, white matter demyelination, and synaptic degeneration, which contribute to the cognitive decline and neuropathological progression of VCID.

### 2.1. Vascular Dysfunction and Cerebrovascular Integrity Loss in VCID

In VCID, accumulating evidence indicates that cerebrovascular pathology is not a secondary correlate of cognitive decline but a primary upstream driver [1,28,29,30]. Both preclinical [31,32] and clinical [33] studies demonstrate that microvascular rarefaction and BBB disruption emerge before neuronal loss and correlate strongly with cognitive deficits, positioning vascular injury as a mechanistic driver of dementia.

Increasing evidence shows that hypertension induces a stereotyped “vascular phenotype”, characterized by endothelial senescence, arterial stiffening, and maladaptive structural remodeling that resembles premature vascular aging. Indeed, chronic high blood pressure accelerates endothelial senescence and microvascular rarefaction, thereby reducing cerebral blood flow and impairing neurovascular coupling [34]. BBB breakdown detectable even in the early stages of cognitive decline occurs independently of amyloid-β and tau deposition [35]. Arterial changes in young hypertensive patients have been shown to mimic those in old individuals who are normotensive [36]. Importantly, antihypertensive interventions reduce the progression from mild cognitive impairment (MCI) to vascular dementia [37,38], reinforcing the concept that correcting vascular dysfunction modifies disease trajectory, not merely symptoms.

Metabolic disorders such as obesity, diabetes, and hyperlipidemia also promote this vulnerability through systemic inflammation, oxidative stress, and lipid dysregulation. Obesity induces BBB leakage and neuroinflammation by downregulating tight junction proteins (ZO-1, occludin, and claudin 5) in brain microvessels [39]. Chronic hyperglycemia in type 2 diabetes promotes endothelial oxidative stress and inflammation that weaken tight junctions, increase transcytosis and BBB permeability, impair oligodendrogenesis and white matter repair after ischemic injury [40,41]. Likewise, dyslipidemia fosters endothelial dysfunction, atherogenic remodeling, and lipotoxic damage to microvessels, leading to reduced capillary density and diminished cerebral microcirculatory reserve, thereby contributing to small vessel disease and progression of white matter hyperintensities [42]. Their convergence on microvascular injury highlights that the vascular network links heterogeneous systemic insults to cognitive decline.

Together, these findings shift the framework for VCID from descriptive vascular abnormalities to a coherent mechanistic model: systemic vascular and metabolic stressors converge on endothelial dysfunction, which then initiate and sustain BBB breakdown, microvascular failure, and neurovascular unit collapse, underscoring the importance of restoring cerebrovascular integrity is central to preventing or delaying VCID progression.

### 2.2. Endothelial Senescence as a Mechanistic Driver of VCID

Rather than serving as a passive marker of aging, accumulating evidence positions vascular senescence as an active driver of VCID. The vascular endothelium forms the innermost layer of blood vessels and plays a vital role in regulating hemostasis and permeability. Continuous endothelial regeneration is essential for maintaining vascular integrity and overall cerebrovascular health. EPCs contribute to this regenerative process by directly differentiating into endothelial cells and releasing paracrine substances [43,44]. Vascular risk factors, such as hypertension, diabetes, and aging, accelerate both endothelial and EPC senescence, diminishing the vascular systems’ capacity for repair [45]. This regenerative reserve aligns with the early appearance of microvascular rarefaction and BBB leakage in VCID. Senescent endothelial cells exhibit reduced NO production and impaired vasodilation responses [46,47], coupled with increased expressions of cytokines and adhesion molecules that enhance leukocyte recruitment and vascular inflammation [46]. Furthermore, disorganization of tight junctions in senescent endothelial cells leads to BBB breakdown [48,49], which is a hallmark of VCID. Additionally, senescent endothelial cells lose their supportive interactions with pericytes and astrocytes, which are essential components of the neurovascular unit, further aggravating BBB disruption. It also contributes to microvascular rarefaction, the progressive loss of capillaries in the brain, which exacerbates cerebral hypoxia, oxidative stress, and neurodegeneration [50,51].

Recent advances highlight mitochondrial dysfunction as a pivotal mechanism linking vascular aging to neurovascular impairment. Growing evidence shows that mitochondria dysfunction promotes endothelial senescence and dysfunction [52]. Endothelial senescence is associated with impaired mitochondrial biogenesis, reduced mitochondrial mass, and altered expression of mitochondrial structural proteins [53]. Deficiency of mitochondrial antioxidant enzymes, such as manganese superoxide dismutase, increases oxidative stress and accelerates endothelial dysfunction. This mitochondrial-driven endothelial injury contributes to chronic hypoperfusion and white matter degeneration, which are core pathological features of VCID.

Collectively, these maladaptive processes form a self-perpetuating cycle in which endothelial senescence and mitochondrial dysfunction amplify vascular injury, BBB breakdown, and neurovascular uncoupling. These pathologies can ultimately lead to white matter demyelination, the core pathological substrates of VCID.

### 2.3. White Matter Damage in VCID

White matter injury is widely recognized as one of the core pathological features of VCID [54], yet its mechanistic underpinnings remain incompletely resolved. In Alzheimer’s disease, genes associated with myelination pathways are found to be the most changed group during the early stages of disease progression [55], suggesting that white matter disruption might be a shared mechanism across neurodegenerative disorders. A key question driving current research is why white matter is disproportionately affected by vascular injury. Its unique anatomical and metabolic features, sparse collateral circulation, long myelinated axons with high energetic demands, and sensitivity of oligodendrocyte lineage cells to hypoxic stress, making it exceptionally vulnerable to perturbations in cerebral perfusion [56]. As cerebrovascular dysfunction accumulates, chronic hypoperfusion and impaired BBB breakdown compromise white matter structure and function [57,58]. Rather than acting independently, these vascular abnormalities interact in a coordinated manner. Loss of cerebrovascular integrity compromises BBB function and cerebral perfusion, triggers inflammatory cascades, and accelerates oligodendrocyte loss and myelin degeneration, ultimately resulting in cognitive impairment [59]. In animal studies, mild chronic hypoperfusion induces vascular damage, oxidative stress and astrogliosis, leading to demyelination, and axonal injury in white matter regions [60]. Consistent with these experimental findings, clinical neuroimaging studies have identified that white matter hyperintensities are strongly associated with hypertension and other vascular risk factors as one of the most robust predictors of cognitive decline and conversion to dementia [59].

**Figure 1:**
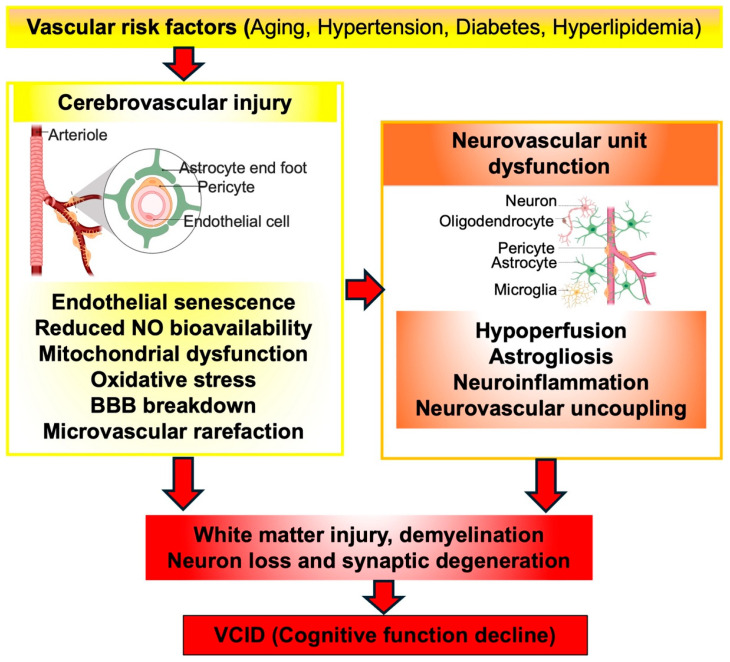
Integrated model of the vascular cascade leading to VCID. Systemic vascular risk factors initiate premature vascular and endothelial senescence, promoting arterial stiffening, oxidative stress, mitochondrial dysfunction, and loss of BBB integrity. These cerebrovascular alterations destabilize the neurovascular unit, resulting in chronic hypoperfusion, neurovascular uncoupling, and sustained neuroinflammatory signaling. The combined vascular and glial dysregulation renders white matter particularly vulnerable, leading to demyelination, axonal injury, and neuronal network degradation. These pathological changes converge to produce the cognitive decline defining VCID. NO: nitric oxide; BBB: blood–brain barrier. “Vessel Icon” created by BioRender, https://www.biorender.com/icon/vessel-generic-branching-1-arteriole-2 (accessed on 20 October 2025); “Astrocyte Icon” created by BioRender, https://www.biorender.com/icon/astrocyte-with-end-feet-1 (accessed on 20 October 2025); “Neuron Icon” created by BioRender, https://www.biorender.com/icon/multipolar-neuron-motor-myelinated-curved-1 (accessed on 20 October 2025); “Microgila Icon” created by BioRender, https://www.biorender.com/icon/microglia-resting-01 (accessed on 20 October 2025).

Taken together, premature endothelial aging amplified by mitochondrial dysfunction impairs vasoreactivity, weakens tight-junction architecture, and promotes a pro-inflammatory, permeability-enhancing phenotype that destabilizes the BBB. As this dysfunction accumulates, microvascular rarefaction and chronic hypoperfusion emerge, creating a metabolic and inflammatory milieu that disproportionately injures vulnerable white matter tracts. The resulting demyelination, axonal degeneration, and neurovascular uncoupling constitute the structural foundation for cognitive decline in VCID. Figure 1 integrates these interactions, highlighting how vascular risk exposure drives a progressive trajectory from endothelial dysfunction to BBB breakdown and ultimately to white matter degeneration and neurocognitive impairment.

### 2.4. Current Treatment Strategies and Unresolved Therapeutic Gaps in VCID

Although VCID imposes a substantial clinical and societal burden, therapeutic progress has been slow. The major limitation is that current management approaches primarily target systemic vascular risk factors rather than directly target the cerebrovascular and neurovascular mechanisms driving cognitive decline. Management of hypertension, diabetes, and dyslipidemia reduces incident VCID and slows its progression, underscoring the causal contribution of vascular dysfunction. However, clinical outcomes vary widely. Pharmacologic interventions such as statins or glucose-lowering therapies yield inconsistent cognitive benefits [61]. The inconsistency across studies reflects unresolved questions about timing, disease stage, and whether systemic normalization of metabolic variables is sufficient to reverse established endothelial senescence, microvascular rarefaction, or BBB instability. Evidence from the Memory and Cognition in Decreased Hypertension sub-study of the Systolic Blood Pressure Intervention Trial (SPRINT-MIND) demonstrates that aggressive blood pressure lowering (<120 mmHg) attenuates white matter hyperintensity accumulation and reduces transition to mild cognitive impairment [25,62,63]. These findings highlight that vascular injury is modifiable, but they also reveal an important constraint: even optimal systemic control does not fully halt white matter degeneration or cognitive decline. This suggests that once cerebrovascular integrity is compromised, downstream neurovascular and white matter pathologies may evolve independently of peripheral vascular metrics.

Additional pharmacological strategies have sought to target oxidative stress, neuroinflammation, or synaptic dysfunction. While conceptually aligned with VCID, these interventions largely mitigate symptoms rather than intervene at the upstream vascular processes that initiate disease [24]. Their modest efficacy illustrates the gap between mechanistic insights from preclinical models and clinically actionable therapies. Understanding endothelial aging, mitochondrial dysfunction, and neurovascular uncoupling has not been investigated by therapies capable of restoring cerebrovascular health.

Collectively, these observations define a major therapeutic gap that no current therapy directly targets the cellular and molecular processes governing vascular resilience, BBB integrity, or neurovascular coupling. This conceptual gap motivates growing interest in regenerative and biologically informed therapies. Stem and progenitor cell-derived EVs represent one such emerging approach. By modulating endothelial function, promoting BBB repair, and restoring neurovascular homeostasis, EV-based therapies may address the upstream drivers of VCID pathology that conventional risk-factor management cannot adequately reverse.

## 3. EVs: Biogenesis, Isolation, and Biomedical Application

EVs represent a heterogeneous communication system used by virtually all cell types, enabling the transfer of bioactive cargo across local and distant tissue compartments. EVs include three major subclasses: exosomes (EXs, diameter: <150 nm), microvesicles (MVs, diameter: 100–300 nm), and apoptotic bodies (diameter: <1000 nm). Their ability to transport proteins, lipids, miRNAs, long non-coding RNAs, and mitochondrial components positions EVs as dynamic mediators of intercellular signaling, particularly relevant to diseases such as VCID. Notably, EV cargo composition mirrors the physiological state of the originating cells, providing both mechanistic insight into disease processes and opportunities for therapeutic modulation. These properties have fueled growing interest in EVs as both biomarkers and therapeutic agents in cerebrovascular disorders.

### 3.1. Biogenesis and Isolation of EVs

Although the fundamental pathways governing EV formation are increasingly well characterized, important mechanistic questions remain unresolved, particularly regarding how cellular stress or senescence alters vesicle composition and release. EX biogenesis involves endosomal maturation and multivesicular body (MVB) fusion with the plasma membrane, whereas MV formation relies on cytoskeletal remodeling, membrane curvature changes, and phospholipid redistribution [64]. Disentangling how these pathways are selectively engaged under pathological conditions, including oxidative stress, endothelial dysfunction, or inflammatory activation, remains a critical area of investigation, especially for understanding EV-mediated signaling in VCID.

A major challenge in EV research is the absence of a single isolation method that consistently balances yield, purity, and preservation of functional integrity across biological samples. Differential ultracentrifugation and density-gradient centrifugation remain widely used for biofluids and cell culture supernatants, offering good yield with moderate purity. Size-exclusion chromatography enables rapid, gentle isolation with high structural integrity and minimal protein and lipoprotein contamination, ideal for proteomic or functional assays [65]. Size-exclusion chromatography, for example, offers gentle isolation well suited for proteomic and functional studies, though its throughput can be limiting in large clinical cohorts. Affinity-based EV enrichment has emerged as a powerful strategy for dissecting cell type specific vesicle populations. Immunocapture targeting neuronal [66] or endothelial [67] markers enables isolation of rare EV subsets from plasma, facilitating biomarker discovery and insights into tissue-specific signaling. However, variability in marker expression and potential loss of non-marker-expressing EVs continue to challenge the interpretation of immunocaptured EV profiles. Technologies such as ExoChip, ExoDisc, and ExoTIC demonstrate how miniaturization and fluidic engineering can reduce sample requirements, enhance reproducibility, and streamline downstream molecular profiling. The ExoChip platform uses surface-immobilized antibodies (e.g., anti-CD63) in a microfluidic channel to selectively capture EVs from small serum or plasma volumes, enabling downstream fluorescent detection and quantitative analysis [68]. The ExoDisc system utilizes a centrifugal microfluidic disc integrated with nanofilters to isolate EVs rapidly from clinical samples with high yield and reproducibility, making it compatible with point-of-care workflows [69]. Similarly, the ExoTIC (Exosome Total Isolation Chip) employs nanoporous membrane filtration to efficiently isolate nanoscale vesicles while preserving their RNA cargo, enabling high-quality transcriptomic analysis from low-input samples [66]. These systems illustrate a broader trend: EV isolation is moving toward precision platforms that better accommodate low-volume clinical samples and support standardized, scalable diagnostics. Yet, the field still lacks consensus benchmarks for comparing methods, underscoring the need for harmonized protocols that can support both mechanistic studies and translational applications in VCID research.

### 3.2. Potential Biomedical Applications of EVs

EVs have gained considerable attention as mediators of disease progression and candidates for therapeutic intervention across neurological and vascular diseases. Their cargo composition shaped by cellular stress, senescence, or metabolic state provides mechanistic insight into disease pathways that are otherwise inaccessible in vivo.

One emerging area of interest is the use of brain-derived EVs or neuron-derived EVs as a minimally invasive “liquid biopsy” for neurodegenerative and neurovascular disorders [67]. Disease-specific alterations in their protein and RNA cargo, such as altered neurofilament light chain, tau, amyloid-β, α-synuclein, and synaptic proteins, which correlate with clinical severity [70], suggesting potential utility in early detection and stratification. MiRs carried by EVs are of particular interest due to their regulatory potency and disease specificity. Clinical studies have demonstrated alterations of EV-derived miRs in Alzheimer’s disease [71,72] and other neurodegenerative conditions. Several miRs, such as miR-223, show associations with cognitive and functional outcomes [73,74], while EV-derived miR-193b correlates with amyloid-β42 levels in cerebrospinal fluid, linking EV cargo directly to Alzheimer’s pathology. Moreover, cerebrovascular endothelial-derived EVs are elevated in individuals with MCI, the earliest symptomatic stage of VCID [75], supporting their utility as early biomarkers of cerebrovascular dysfunction. However, challenges remain in isolating truly cell-type-specific vesicle populations and distinguishing disease-driven cargo changes from confounding systemic factors. These limitations underscore the need for methodological standardization before EV-based biomarkers can be integrated into VCID diagnostics.

EVs also participate directly in the biology of vascular aging and cerebrovascular injury. Vesicles released by senescent vascular cells or damaged vascular tissue propagate endothelial dysfunction, vascular calcification, and inflammation, exacerbating cerebrovascular pathology [76]. Conversely, EVs from young or healthy cells can convey pro-reparative molecular programs. Studies revealed that circulating EVs isolated from young mice can reverse the transcriptional alterations of the aged mouse pulmonary vasculature, leading to a more youthful endothelial transcriptional phenotype [77]. Adipose-derived MSC-EVs reduce ROS production in vascular endothelial cells and upregulate C1q/TNF-related protein 9, an anti-inflammatory and anti-senescent factor downregulated during vascular aging [78]. These findings support the concept that EV cargo is not merely reflective of cellular state but can actively reshape vascular homeostasis.

Beyond their mechanistic roles, EVs are increasing viewed as potential therapeutic platforms capable of restoring cerebrovascular function. Preclinical studies show that administration of stem and progenitor cell-derived EVs can restore BBB integrity, enhance mitochondrial biogenesis, and attenuate neuroinflammation. EPC-derived EVs enriched in miR-126 promote endothelial regeneration and collateral vessel formation following ischemic stroke [13], while MSC-EVs improve neurocognitive recovery and reduce neuroinflammation in aged mice [79]. Recent studies further demonstrate that hypoxia-preconditioned MSC-EVs enhance tight-junction protein expression (ZO-1, occludin) and reduce MMP-9 mediated BBB degradation [80]. Similarly, EPC-EVs repair ischemic brain microvasculature and improve perfusion by activating the PI3K/Akt/eNOS signaling pathway [81]. Despite these encouraging findings, critical challenges remain notably in defining dose, biodistribution, cargo variability, and long-term safety, underscoring the need for more rigorous translational frameworks.

Collectively, these observations position EVs at the intersection of diagnostics, mechanistic biology, and regenerative therapy. Their ability to traverse the BBB, modulate endothelial signaling, and deliver functional miRs and proteins makes them uniquely suited for addressing the upstream vascular and neurovascular deficits characteristic of VCID. The following sections focus on stem- and progenitor cell-derived EVs, which show promise for repairing cerebrovascular damage and mitigating cognitive decline in VCID.

## 4. Stem Cells and Their Derived EVs in VCID

Stem and progenitor-like cells, including EPCs, MSCs and pericytes, play critical roles in maintaining vascular homeostasis and orchestrating reparative responses after injury. EPCs support endothelial regeneration both through directly differentiating into endothelial cells and paracrine signaling [82,83,84]. Impairment or loss of EPC function intricately linked with defective endothelial repair and greater susceptibility to VCID and small vessel diseases. Similarly, MSCs possess potent anti-inflammatory, anti-apoptotic, and pro-angiogenic effects that benefit ischemic and neurodegenerative conditions largely through their secretomes. Pericytes which tightly envelop endothelial cells, are essential for maintaining capillary integrity and BBB stability, representing another critical yet vulnerable component of the cerebrovascular architecture. Their degeneration is implicated in small vessel disease and white matter injury [82]. A major conceptual advance in recent years is the recognition that many of the reparative effects attributed to EPCs, MSCs, and pericytes are mediated through their EVs. The shift from a “cell therapy” to an “EV therapy” paradigm has broad implications for VCID research, offering a more stable, scalable, and mechanistically targeted approach to restoring cerebrovascular function.

### 4.1. Possible Roles of EPCs and Their Derived-EVs in VCID

EPCs possess strong regenerative and angiogenic potential. They are considered promising candidates for cerebrovascular diseases. Both our group and others have shown that EPC transplantation enhances angiogenesis [81] and vascular remodeling in ischemic brain injury models [85]. Nevertheless, EPC function is highly sensitive to vascular risk factors. Hyperlipidemia, hyperglycemia, hypertension, and smoking accelerate EPC senescence, reduce their proliferative capacity, and impair NO-dependent angiogenic signaling. For example, high-glucose conditions induce EPC senescence by inhibiting the SIRT1/PI3K/Akt/eNOS pathway [86]. Salt-sensitive hypertension decreases telomerase activity in EPCs [87], and cigarette smoke extract suppresses eNOS expression while elevating p16 and apoptosis, thereby impairing EPC angiogenic function [88]. Clinical evidence shows the correlation between reduced circulating EPC numbers and cognitive impairment in patients with Alzheimer’s disease [89]. Shimizu et al. demonstrated that vascular endothelial stem cells from aged mice exhibit reduced colony-forming and proliferative potential compared with those from young mice, both in vitro and in vivo [90]. Similarly, senescent human EPCs display downregulation of CCL5, a chemokine critical for EPC homing and angiogenesis [91]. These findings suggest that EPC exhaustion represents an early event linking vascular risk exposure to impaired endothelial repair capacity.

Increasing evidence shows that EPCs exert their reparative actions through the secretion of EVs. Deregibus et al. first demonstrated that EPC-derived MVs trigger angiogenesis by horizontally transferring mRNA to vasculature endothelial cells [92]. Another study showed that umbilical cord blood EPC-EXs promoted angiogenesis of human microvascular endothelial cells via activation of the Erk1/2 signaling pathway [93]. Ke et al. reported that endothelial colony-forming cell-derived EXs carrying miR-21-5p enhanced human microvascular endothelial cell viability and proangiogenic capability in an in vitro atherosclerosis model and in vivo rat study [94]. Hu and colleagues found that EPC-EXs improved vascular reendothelialization in rats [95,96]. Our group has shown that EPC-EXs enriched in miR-126 markedly decrease ischemic injury and preserve cerebral blood flow in diabetic stroke mice [13]. These effects are accompanied by enhanced angiogenesis, neurogenesis, and improved neurological recovery [13]. Other studies corroborated our findings and showed that EPC-EXs reduced infarct size and neuronal apoptosis via downregulating Wnt3 and phosphorylated GSK-3β [97]. EPC-MVs produced under nutrient deprivation have been shown to promote endothelial survival and function through the PI3K/Akt pathway [98]. In addition, EPC-EVs can modulate microglial polarization, shifting cells from the pro-inflammatory M1 to the anti-inflammatory M2 phenotype, thereby reducing neuroinflammation and secondary white-matter injury in a spinal cord injury mouse model [99]. EPC-EVs also exert protective effects on mitochondria. In a model of endothelial oxidative stress, EPC-EVs delivered functional mitochondrial components that restored mitochondrial membrane potential and reduced cytochrome-c release [100]. This mitochondrial resilience may represent a key therapeutic advantage of EPC-EV-based approaches.

Taken together, these findings demonstrate the multifaceted reparative capacity of EPC-EVs. By delivering pro-angiogenic, anti-inflammatory, and mitochondrial-stabilizing cargo, EPC-EVs directly counteract vascular dysfunction, BBB disruption, and neurovascular unit impairment that drive VCID progression. Their ability to reestablish neurovascular homeostasis and improve neurological outcomes in preclinical models provides a compelling rationale for advancing EPC-EVs toward translational development in VCID.

### 4.2. Possible Roles of MSCs and Their Derived EVs in VCID

MSCs have been widely explored as therapeutic candidates in VCID due to their ability to modulate inflammation, enhance angiogenesis, and support neurovascular repair. Using a bilateral carotid artery stenosis (BCAS) model of vascular dementia, Kim and colleagues demonstrated that treatment with human embryonic stem cell-derived multipotent MSCs significantly improved both working and long-term memory in mice [101]. This cognitive improvement was accompanied by the restoration of tight junction proteins (claudin-5, occludin), angiogenic proteins (Ang1, TieI, and TieII), and neuron markers (Neurod, Cux), along with reduced astrocytic activation [101]. Similarly, Lee et al. showed that MSC administration enhanced spatial working memory in 5xFAD transgenic Alzheimer’s mice subjected to BCAS [102]. In a cerebral small vessel disease model, MSCs improved learning ability and cognitive performance by repairing the BBB and mitigating brain atrophy [103].

Interestingly, MSC-EVs recapitulate many of the parent cells’ protective functions. EXs from human umbilical cord MSCs promote proliferation and migration of human umbilical vein endothelial cells via the Wnt4-mediated activation of the Wnt/β-Catenin signaling pathway [104], while concurrently inhibiting COX-2 expression to suppress inflammatory and cellular senescence [105]. Shabbir et al. demonstrated that MSC-EVs enhance endothelial angiogenesis through activation of the STAT3 signaling [106], while Anderson et al. identified a plethora of proteins in human MSC-EXs, highlighting their complex bioactive cargo [107]. Notably, EVs from young MSCs, but not aged ones, can rejuvenate senescent EPCs in mice. The aged MSC-EVs carry higher levels of miR-146a, miR-10a∗, miR-21, and miR-29c, but reduced levels of pro-angiogenic miR-126 [108]. In ischemic and hypoperfusion models relevant to VCID, MSC-EVs consistently demonstrate neurovascular protection. Administration of MSC-EVs after middle cerebral artery occlusion or focal cerebral ischemia reduces infarct volume, enhances motor and behavioral recovery, increases NeuN+ neuronal density, and promotes neurogenesis [109,110,111,112,113]. EXs derived from miR-26a-modified MSC could improve neurogenesis, promote axonal regeneration and attenuate astrogliosis through PTEN/AKT/mTOR signaling cascades [114]. EXs released from adipose derived MSCs similarly alleviate brain edema and support neurological recovery in acute stroke [115]. Moreover, bone marrow-derived MSC-EVs protect baroreceptor reflex function following hypoxic–ischemic injury, suggesting sustained protection of brainstem autonomic circuits [116]. Importantly, recent studies directly implicate MSC-EVs in VCID-like pathology. In a BCAS-induced VCID mouse model, MSC-EXs restored cognitive and synaptic function, reduced neuronal injury, and attenuated Aβ protein accumulation and Tau hyperphosphorylation by regulating the Ras/Akt/GSK-3β pathways [117]. These effects are consistent with findings in Alzheimer’s disease models, where MSC-EVs decrease inflammatory cytokine expression, promote Aβ degradation, improve glucose metabolism, inhibit astrocyte reactivity, and enhance synaptic plasticity [118,119,120,121]. In addition to primary MSC-derived EVs, immortalized human MSC lines have been used as in vitro and preclinical models to investigate EV-mediated vascular and neuroprotective mechanisms. EVs derived from immortalized adipose tissue-derived MSCs have been shown to promote endothelial cell proliferation and enhance angiogenic properties in dermal endothelial cells, supporting a conserved pro-angiogenic EV signaling profile across MSC sources [122]. Furthermore, in neonatal mouse models of hypoxic–ischemic brain injury, immortalized human MSC-EVs derived from reduced post-ischemic brain atrophy, neuronal loss, and vascular injury. Notably, these vascular-protective effects were associated with suppression of vascular cell adhesion molecule-1 upregulation and reduced leukocyte adhesion, alongside restoration of endothelial cell proliferative capacity [123]. Together, these findings underscore the broad neurovascular benefits of MSC-EVs and their promising potential as a therapeutic strategy to mitigate cerebrovascular damage and cognitive decline in VCID. Of note, important questions remain regarding optimal dosing, biodistribution, and standardization of EV production issues that will be critical to address as the field moves toward clinical testing.

### 4.3. Potential Roles of Pericytes and Their Derived EVs in VCID

Pericytes are mural cells that wrap around microvessels and play essential roles in microvascular stability and BBB integrity [124,125,126]. A substantial body of work have suggested that subsets of pericytes exhibit mesenchymal stem/progenitor-like properties [12], including multipotency and self-renewal capacity, based on shared marker expression and differentiation potential observed under defined experimental conditions. These studies have reported pericyte differentiation into mesenchymal and neural-associated lineages, including chondrocytes [127], adipocytes [127], osteoblast-like cells [128], neural progenitor-like cells [129], vascular cells [129], and microglia-like phenotypes [130] in vitro and in selected in vivo injury models. In contrast, lineage-tracing and fate-mapping studies in uninjured adult tissues indicate that pericytes do not universally function as bona fide MSCs in vivo, highlighting substantial heterogeneity among pericyte populations and context-dependent plasticity [131]. Importantly, regardless of their debated stem/progenitor status, pericytes are indispensable regulators of vascular aging, BBB integrity, and neurovascular unit stability, processes central to VCID pathogenesis. Accordingly, this review focuses primarily on pericytes and their secreted EVs as key modulators of cerebrovascular integrity, neurovascular communication, and white matter vulnerability in VCID.

Growing evidence indicates that pericyte loss or dysfunction is increasingly recognized as a key pathogenic event in VCID [124,125,132]. Ding et al. (2020) reported a 35–45% reduction in collagen type IV-positive pericytes in the deep frontal white matter of VCID patients, and a subsequent study in 2024 found decreased pericyte density per capillary length in the hippocampal CA1 region [133,134]. These findings parallel those in Alzheimer’s disease, where pericyte degeneration precedes neuronal loss and correlates with BBB breakdown [135]. It has previously been shown that chronic cerebral hypoperfusion, resulting in decreased pericyte coverage in the corpus callosum and cortex of mice on day 3 post-infarct, is followed by gradual recovery until cognitive dysfunction develops on day 30 [136]. The decrease in COLA4+ pericytes in the frontal deep white matter of those with VCID was similarly associated with elevated fibrinogen levels and decreased PDGFRβ expression, linking pericyte depletion to BBB leakage [133,137]. Importantly, pericyte loss or functional decline contributes not only to the BBB leakage but also to neurovascular uncoupling, a central mechanism underlying cognitive impairment in VCID [138,139,140].

Pericytes, like endothelial cells, are vulnerable to cellular senescence under conditions of vascular stress. Transcriptomic profiling of senescent pericytes reveals upregulation of senescence-associated cytokines (e.g., IL-6) [141] and extracellular matrix genes (COL18), along with reduced PDGF signaling [142]. In aged mouse models, senescent pericytes impaired BBB function [143,144]. Endothelin-1 has also been linked to senescence of brain microvascular pericytes under diabetic conditions in vitro [145]. Collectively, these studies suggest that pericyte senescence links vascular risk factors and chronic BBB dysfunction in VCID.

Given their dynamic role in maintaining cerebrovascular homeostasis, pericytes also communicate through EVs (pericyte-EVs). Pericyte-EVs carry miRs, such as miR-143, miR-26a, miR-122-5p, miR-21, and miR-181a, many of which are implicated in cerebrovascular disease, endothelial activation, and neurodegeneration [124]. Several of these miRs (notably miR-143 and miR-21) regulate SMAD2/3-TGFβ, PI3K/Akt, and NF-κB pathways, suggesting that pericyte-derived EVs can influence vascular remodeling and inflammatory responses. Conversely, several studies highlight the reparative potential of pericyte-EVs. Under hypoxic conditions, hypoxia-inducible factor (HIF-1a) activation in human brain pericytes enhance wound healing and angiogenesis via EV-mediated paracrine signaling [146]. Guo et al. demonstrated that EVs are involved in enhanced revascularization through pericyte-endothelial interactions in a mouse model of ischemic injury [147]. Likewise, brain microvascular pericyte-EVs from spontaneously hypertensive Wistar Kyoto rats exhibited substantial remodeling of the miRs (17 miRs upregulated and 31 downregulated miRs) [148]. Many of these miRs are predicted to modulate pathways such as α-linolenic acid and sphingolipid metabolism, both of which are involved in hypertension-related vascular pathology. Thus, pericyte-EVs may function as mediators of neurovascular injury, representing a novel therapeutic strategy for VCID.

In addition to adult stem cell-derived EVs, induced pluripotent stem cell (iPSC)-derived EVs are gaining attention for their therapeutic potential in vascular and neurovascular disorders. iPSC-EVs have demonstrated beneficial effects on endothelial function, including reversal of glucose-induced reductions in cell viability, attenuation of endothelial senescence, and preservation of capillary structural integrity in human umbilical vein endothelial cells. Moreover, iPSC-EVs have shown protective effects on BBB integrity in ischemic stroke models, including aged mice. Notably, Li and colleagues reported that iPSC-derived small EVs reduce BBB senescence and restore the expression of tight junction proteins such as claudin and ZO-1 [149]. While these findings underscore the therapeutic promise of iPSC-EVs for vascular and BBB repair, their biological mechanisms, manufacturing considerations, and translational challenges have been comprehensively reviewed elsewhere [150].

**Figure 2:**
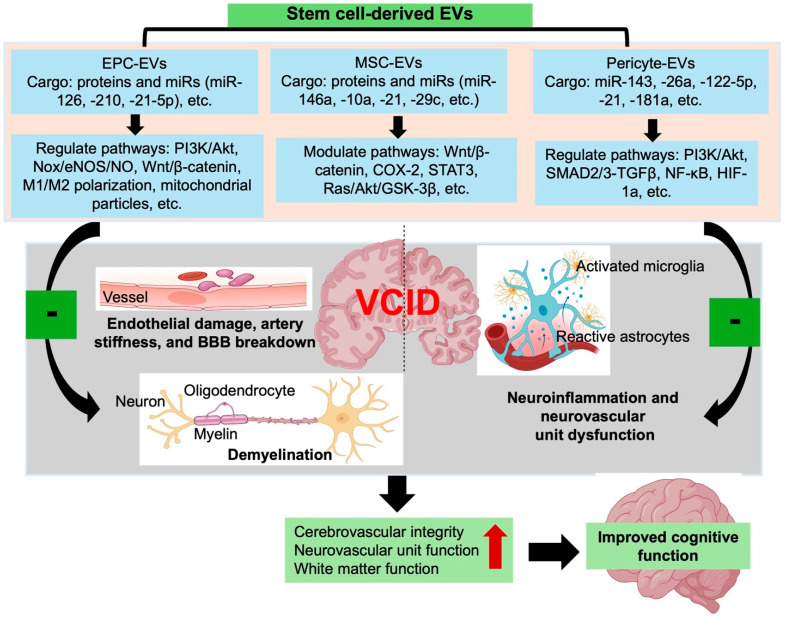
Therapeutic actions of stem cell–derived EVs in VCID. EVs derived from EPCs, MSCs, and pericytes carry distinct yet complementary cargo that modulate angiogenic, anti-senescent, anti-inflammatory, and neuroprotective pathways. By promoting BBB repair, restoring endothelial and pericyte function, suppressing glial activation, and supporting myelination, these EVs collectively counteract neurovascular unit dysfunction and white matter injury, ultimately improving cognitive outcomes in VCID. “Brain Icon” created by BioRender, https://www.biorender.com/icon/brain-coronal-cut-at-basal-ganglia accessed on 20 October 2025); “Reactive Astrocyte Icon” created by BioRender, https://www.biorender.com/icon/astrocyte-with-blood-vessel-reactive accessed on 20 October 2025).

Taken together, EVs derived from EPCs, MSCs, and pericytes collectively highlight a convergent therapeutic paradigm in which cell-free vesicular signaling targets the core vascular and neurovascular deficits underlying VCID. Despite their distinct cellular origins, these EV populations share the ability to counteract endothelial senescence, enhance angiogenic and reparative signaling, stabilize the BBB, and modulate inflammatory and glial responses that drive neurovascular unit dysfunction. By coordinating protective effects across multiple components of the cerebrovascular system, these vesicles offer a mechanistically integrated strategy to restore vascular homeostasis, preserve neurovascular coupling, and prevent the progression of white matter injury. Figure 2 summarizes the complementary and intersecting roles of EPC-, MSC-, and pericyte-derived EVs in safeguarding cerebrovascular function and maintaining the integrity of the neurovascular unit.

## 5. Conclusions and Future Perspectives of Stem Cell-Derived EVs for VCID Treatment

Stem cell-derived EVs from EPCs, MSCs, and pericytes represent a compelling acellular therapeutic strategy for VCID. These vesicles recapitulate reparative properties of their parent cells, such as angiogenic and endothelial repair, BBB stabilization, neuroinflammation mitigation, and white-matter restoration, while offering advantages such as lower immunogenicity, greater stability, and the capacity to cross the BBB. Particularly, EPC-EVs enhance vascular integrity through pro-angiogenic and mitochondrial protective cargo; MSC-EVs deliver diverse regenerative and anti-apoptotic signals that support synaptic and cognitive recovery; and emerging evidence suggests that pericyte-derived EVs may stabilize the neurovascular unit. Together, these findings highlight these EVs as a versatile platform capable of targeting multiple pathological hallmarks of VCID.

Despite substantial progress, several major gaps hinder translation. The mechanistic specificity of EV cargo remains poorly defined; EV heterogeneity driven by the cell source, donor age, culture conditions, and the isolation method poses significant challenges for reproducibility. Fundamental questions regarding optimal dosing, biodistribution, route of delivery, and long-term safety remain unanswered. Furthermore, comorbid conditions common in VCID populations, such as hypertension, diabetes, and hyperlipidemia, likely influence EV biogenesis and therapeutic potency, yet these interactions are not well understood.

A critical future direction is to delineate the dualistic role of EVs in VCID. EVs produced under pathological conditions may exacerbate vascular injury by transporting pro-inflammatory, pro-senescent, and barrier-disruptive cargo that accelerates endothelial aging and white matter degeneration. In contrast, EVs from young or healthy cells appear to possess unique rejuvenate properties capable of enhancing endothelial resilience, stabilizing BBB, modulating microglial and astrocytic activation, and promoting oligodendrocyte maturation. Determining whether “youthful” EVs can meaningfully slow or reverse VCID progression and identifying the cargo responsible for these effects represents a high-priority research frontier.

Advances in multi-omics profiling, single-EV analysis, and EV bioengineering provide powerful opportunities to define causal bioactive components and develop next-generation EVs with enhanced targeting and therapeutic precision. Integrating these technologies with rigorous preclinical modeling will help transition stem cell-derived EVs from promising experimental tools to clinically actionable therapies for VCID.

Besides the above-mentioned EVs, neural stem cell-derived EVs have also been investigated in central nervous system disease models such as ischemic stroke [151], acute brain injury [152], and neuroinflammation [153]. NSC-derived EVs have been shown to reduce neuronal apoptosis, modulate microglial activation, and support angiogenic and reparative signaling in ischemic contexts. However, direct evidence evaluating NSC-derived EVs in VCID or chronic cerebral hypoperfusion models remains limited. While NSC-derived EVs represent a promising therapeutic avenue, their specific roles in cerebrovascular dysfunction in VCID remains to be systematically explored.

## Data Availability

No new data were created or analyzed in this study.
